# Meeting of the Texas State Medical Association

**Published:** 1895-04

**Authors:** 


THE MEETING of the Texas State Medical Association at Dallas, beginning fourth Tuesday this month, April, promises to be unusually full of interest, judging from the very elaborate program published herewith; and it is especially gratifying to learn, as we do, that preparation is being made by the reception committee and citizens, to entertain delegates in a social way; and further, the indications are that there will be an unusually large attendance and from all parts of the State. The central location of Dallas and the attractions and the low rate of fare insure a large attendance. All this is especially gratifying to the JOURNAL, whose life has been largely devoted to building up and maintaining this organization, and especially demanding a high state of ethics; because, at the Austin meeting, when Dallas was selected by the nominating committee for this meeting, in the absence of any invitation,—something which has never occurred before, to our knowledge,—there were those to say we would not be welcome; that the Association had volunteered to meet at Dallas; had gone there unasked, and that we might expect a turn of that dreaded cold shoulder. The hatchet is buried, and reunited, North, South, East and West Texas, represented by the JOURNAL'S readers, will once more smoke the pipe of peace, and be happy. Let all go who possibly can. The time will be well spent, and none will ever regret it. The JOURNAL will, as usual, publish the proceedings in the May number. All aboard for Dallas!

